# Increased hospitalizations among sarcoidosis patients from 1998 to 2008: a population-based cohort study

**DOI:** 10.1186/1471-2466-12-19

**Published:** 2012-07-09

**Authors:** Alicia K Gerke, Ming Yang, Fan Tang, Joseph E Cavanaugh, Philip M Polgreen

**Affiliations:** 1 Department of Internal Medicine, University of Iowa, 200 Hawkins Drive, Iowa City, IA, 52242, USA; 2 Department of Biostatistics, University of Iowa, 105 River Street, Iowa City, IA, 52242, USA

**Keywords:** Sarcoidosis, Epidemiology, Hospitalizations, Outcomes, Trends

## Abstract

**Background:**

Diagnostic and treatment approaches for sarcoidosis have changed dramatically over the past decade. Yet, the most recent reports of trends in hospitalizations of sarcoidosis patients are over ten years old. The objectives of this study were to determine the incidence of sarcoidosis among hospitalized patients and to analyze recent trends and seasonality of hospitalizations in sarcoidosis patients.

**Methods:**

We performed a retrospective cohort study of the Nationwide Inpatient Sample from 1998 through 2008. We identified all hospitalizations with a primary or secondary diagnosis of sarcoidosis (ICD-9-CM code 135). Incidence was modeled as a seasonal time series about a linear trend.

**Results:**

Time series analysis of the monthly number of hospitalizations revealed a distinct positive linear trend. Over the study period, the number of hospitalized patients with sarcoidosis increased from 37,516 to 70,947 cases. Trends were most pronounced in patients older than 55 years (p < 0.0001), African Americans (p < 0.0001), females (p = 0.0289), and non-Medicaid populations (p < 0.0001). Hospitalizations are seasonal with highest incidence in January through March.

**Conclusions:**

Hospitalizations among sarcoidosis patients have almost doubled during the past decade, with disproportionate rate increases in African Americans, women, and older patients. The rate also increases among patients with insurance other than Medicaid. This study indicates the need for heightened surveillance of sarcoidosis patients given the unknown consequences of evolving treatment approaches. Our results point to a need for research investigating risk factors for hospitalization, including medications, co-morbidities, demographics, and socioeconomic status.

## Background

Sarcoidosis is a systemic granulomatous inflammatory disease of unknown etiology which can cause significant morbidity [1,2]. It is characterized by the presence of non-caseating granulomas, and although it can involve almost any organ system, it primarily affects the lungs [3]. Sarcoidosis affects all races, ages, genders, and ethnicities, but African Americans and women commonly have more severe disease [3,4]. Sarcoidosis is not a reportable disease in the United States (U.S.); therefore, little is known about recent disease trends among patients in the U.S.

Prior studies focusing on hospitalization trends among patients with sarcoidosis report conflicting results. A study of U.S. Navy personnel showed a steep decline in hospitalizations for sarcoidosis over the time period of 1975 to 2001, with the authors concluding that this was due to improvements in protection from occupational exposures that may have been causing sarcoidosis, or reflecting improvement in a ‘sarcoid-like’ disease from these exposures [5]. However, a study using data from the national hospital discharge survey from 1979–2000 demonstrated an increase in hospitalizations; women and African Americans had the highest frequency of hospitalization [6].

Since these studies were published, diagnostic and treatment approaches have changed dramatically. More sensitive imaging technologies to detect and monitor organ involvement are now available, and increased use of immunosuppressive alternatives to prednisone (such as methotrexate, leflunomide, azathioprine, and infliximab) has altered clinical management over recent years [7-13]. Despite changing treatment and clinical strategies, there are no published data reporting hospitalization trends and outcomes in sarcoidosis patients over the past ten years in the United States. This type of epidemiologic information is important to assess the burden of this disease over time, particularly in light of a recent study indicating that overall mortality in sarcoidosis patients may be increasing [14].

The goal of this study was to determine the incidence of sarcoidosis among hospitalized patients and to analyze the recent trend in hospitalizations among patients with sarcoidosis. We also examined demographics, hospital mortality, length of stay, and seasonal disease patterns.

## Methods

All data were extracted from the Nationwide Inpatient Sample (NIS), the largest all-payer database of national hospital discharges in the United States. The purpose of the NIS is to specifically identify, track, and analyze national trends in healthcare utilization, access, charges, quality, and outcomes. It is maintained as part of the Healthcare Cost and Utilization Project by the Agency for Healthcare Research and Quality (AHRQ) and contains data from a 20 % stratified sample of nonfederal acute hospitals [15]. This sample includes academic medical centers, community hospitals, general hospitals, and specialty hospitals. It excludes long-term care facilities and rehabilitation hospitals. To adjust for yearly changes in the sample, we applied the weights provided by the AHRQ [15]. The study investigators obtained approval for use of this database from AHRQ/HCUP by submission of a data use agreement. This is a de-identified dataset and our IRB has determined that this study is not human subjects research. We first identified all hospitalizations during the period from 1998 through 2008 during which a primary or secondary diagnosis of sarcoidosis was received. For case ascertainment, we used the International Classification of Diseases, 9^th^ Revision, Clinical Modification (ICD-9-CM) code 135. We then aggregated all cases of sarcoidosis by month to produce a national sample of cases of sarcoidosis over time. Cases of sarcoidosis were assigned to a calendar month on the basis of the date that the patient was admitted to the hospital. The numbers of sarcoidosis cases were further converted to incidence rates to account for the variation in total overall number of all hospitalized discharges in each month.

The yearly series for hospital mortality rate and average length of stay were also extracted to monitor the characteristics of sarcoidosis patients over time. For each year, the hospital mortality rate is defined as the number of deaths during hospitalization over the total number of sarcoidosis cases in that year.

We performed a time series analysis of the sarcoidosis rate over the study period. Specifically, we built a seasonal autoregressive integrated moving average (SARIMA) model, which accounted for the serial correlation and allowed us to test for a potential trend and for seasonality in sarcoidosis incidence. After obtaining a suitable model for the national data, we then applied the same model structure to each sub-series stratified by the dichotomous variables age (< 55 vs. > = 55), race (black vs. non-black), gender (female vs. male), and payer (Medicaid vs. non-Medicaid). We chose 55 years old as an age cut-off in order to capture the second bimodal peak of presentation of sarcoidosis patients in their 60’s that has been previously noted in epidemiological studies [3]. In a similar fashion, we conducted a time series analysis of hospitalized patients with a primary diagnosis of sarcoidosis based on all the scenarios described above.

To determine if there was a trend in hospital mortality rate, we fit a logistic regression model with the year as the independent variable. Generalized estimating equations were used for parameter estimation and an autoregressive correlation structure was incorporated to account for the temporal association in the outcome. To investigate whether there was a trend in average length of stay, we fit an analogous linear regression model, assuming normality for the distribution of the outcome.

All statistical analyses were performed using R version 2.11.1 (R Foundation for Statistical Computing) and SAS 9.2 (Cary, North Carolina, USA).

## Results

**Figure 1:**
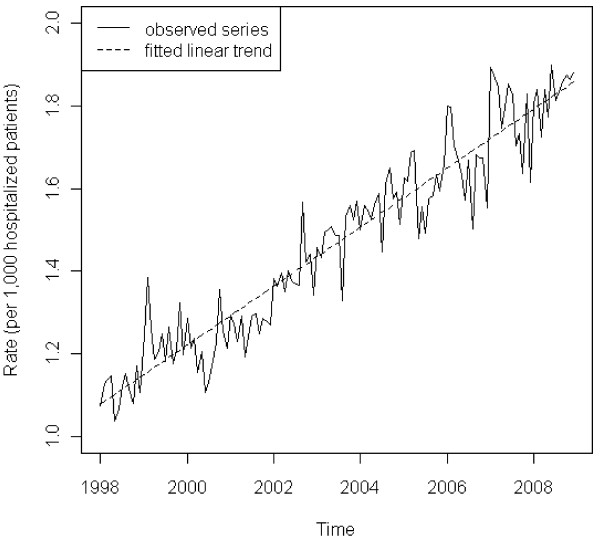
**Monthly incidence rates of hospitalizations among patients with sarcoidosis (Nationwide Inpatient Sample, 1998–2008).** The solid line represents the observed rate over time. The dashed line is the fitted linear trend (p < 0.0001)

Over the study period, the estimated number of hospitalized patients with sarcoidosis almost doubled from 37,516 cases in 1998 to 70,947 cases in 2008. Our time series analysis of the sarcoidosis incidence rate revealed a significant upward trend (Figure 1). Our final SARIMA model contained first-order (p = 0.0155) and second-order (p = 0.0167) moving average terms, a first-order seasonal (period of 12) moving average term (p = 0.0169), and an upward linear time trend (p < 0.0001). Evidence of seasonality was provided by the test result of the seasonal moving average component. Based on the monthly distributions of the incidence rates from the detrended series, it appeared that January, February, and March were the peak months for sarcoidosis hospitalizations. The model showed no lack of fit based on the Durbin-Watson test (p = 0.4872) and an inspection of the autocorrelation function (ACF) and partial ACF of the residuals. The significant increase in the incidence rate was confirmed in all sub-series stratified by age, race, gender, and payer (Table 1).

**Table 1:** Summary of linear trend tests for incidence rates of hospitalizations of patients with sarcoidosis (Nationwide Inpatient Sample, 1998–2008)

	**Trend**			**Difference**		
	**Coef.**	**Std. Err.**	**P-Value**	**Coef.**	**Std. Err.**	**P-Value**
Age: < 55	3.4783	0.3137	< 0.0001*	−5.5510	0.4182	< 0.0001*
Age: > = 55	9.0293	0.2766	< 0.0001*			
Race: Black	19.3509	1.4441	< 0.0001*	15.0865	1.4535	< 0.0001*
Race: Non-Black	4.2644	0.1652	< 0.0001*			
Gender: Female	6.3568	0.3012	< 0.0001*	0.8158	0.3735	0.0289*
Gender: Male	5.5410	0.2208	< 0.0001*			
Payer: Medicaid	1.7254	0.3425	< 0.0001*	−5.2582	0.4372	< 0.0001*
Payer: Non-Medicaid	6.9836	0.2718	< 0.0001*			

The linear time trend (monthly time index / 1000) is included in all models as a deterministic covariate. The difference is defined by the slope in the first category minus the slope in the second category.

* indicates significance at 0.05 level.

**Figure 2:**
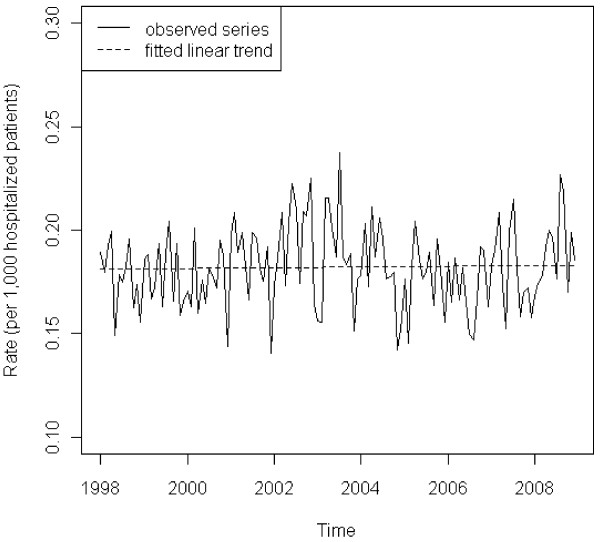
**Monthly incidence rates of hospitalization due to a primary diagnosis of sarcoidosis (Nationwide Inpatient Sample, 1998–2008).** The solid line represents the observed rate over time. The dashed line is the fitted linear trend (p = 0.7259)

The same model structure was applied to analyze the monthly incidence rates due to primary diagnosis of sarcoidosis. The second-order moving average did not appear to be significant and thus was dropped from the model. The seasonal moving average and linear time trend were retained to provide statistical tests for potential seasonality and trend. The first-order moving average (p = 0.0467) remained in the final model, but the first-order seasonal moving average (p = 0.3195) and linear time trend (p = 0.7259) were no longer significant (Figure 2), indicating no observed seasonal variation or upward/downward trend in incidence rates due to primary diagnosis of sarcoidosis. Again, the model showed no lack of fit based on the Durbin-Watson test (p = 0.4973) and an inspection of the ACF and partial ACF of the residuals. Similar results of linear trend tests were observed in sub-series stratified by race, gender, and payer (Table 2). However, for age we observed a significant decrease in the incidence rate (p = 0.0007) among the younger population (i.e., age < 55).

**Table 2:** Summary of linear trend tests for incidence rates of hospitalizations due to a primary diagnosis of sarcoidosis (Nationwide Inpatient Sample, 1998–2008)

	**Trend**			**Difference**		
	**Coef.**	**Std. Err.**	**P-Value**	**Coef.**	**Std. Err.**	**P-Value**
Age: < 55	−0.2604	0.0770	0.0007*	−0.6434	0.0968	< 0.0001*
Age: > = 55	0.3830	0.0587	< 0.0001*			
Race: Black	0.5696	0.3495	0.1032	0.4970	0.3519	0.1579
Race: Non-Black	0.0726	0.0412	0.0780			
Gender: Female	0.0423	0.0698	0.5445	0.0406	0.0992	0.6824
Gender: Male	0.0017	0.0705	0.9808			
Payer: Medicaid	−0.1288	0.1105	0.2438	−0.1776	0.1249	0.1552
Payer: Non-Medicaid	0.0488	0.0583	0.4026			

The linear time trend (monthly time index / 1000) is included in all models as a deterministic covariate.

The difference is defined by the slope in the first category minus the slope in the second category.

* indicates significance at 0.05 level.

We compared the slopes of linear trends between different sub-series stratified by age, race, gender, and payer. For the general sarcoidosis population (Table 1), we observed larger slopes among the older (p < 0.0001), black (p < 0.0001), female (p = 0.0289), and non-Medicaid (p < 0.0001) sub-populations. For patients with a primary diagnosis of sarcoidosis, we also observed larger slopes among the older (p < 0.0001), black (p = 0.1579), female (p = 0.6824), and non-Medicaid (p = 0.1552) sub-populations, although the difference in slopes is only significant for the two sub-series stratified by age.

Over the study period, we found significant downward trends in hospital mortality rate among both the general sarcoidosis population (trend estimate = −0.0359, model-based SE = 0.0080, p < 0.0001) and patients with a primary diagnosis of sarcoidosis (trend estimate = −0.0661, model-based SE = 0.0168, p < 0.0001). A significant decrease in average length of stay was also observed for general sarcoidosis population (trend estimate = −0.0482, model-based SE = 0.0101, p < 0.0001), but not for patients with a primary diagnosis of sarcoidosis (trend estimate = 0.0110, model-based SE = 0.0132, p = 0.4047).

## Discussion

Our results show a dramatic increase in the number of overall hospitalizations of sarcoidosis patients in the U.S. between 1998 and 2008. We found the highest increase in the rate of hospitalizations among African Americans, females, older patients, and those with insurance other than Medicaid. However, for those admitted with sarcoidosis listed as the primary diagnosis, the incidence was stable during the study period with no significant differences in trends between races, gender, or insurance status. Thus, it appears that this increasing trend is driven by admissions for people with sarcoidosis, rather than because of sarcoidosis.

Although sarcoidosis was first described over 100 years ago, the cause is still unknown, treatment is still controversial, and epidemiologic data are difficult to collect due to the rarity of the disease and diverse clinical presentations. Our findings are important as there are few published data on the burden of hospitalizations in this disease in the past ten years. Our results clearly demonstrate that there is an increasing burden of inpatient medical care required by sarcoidosis patients, and that this trend is greater in older patients, women, and African Americans.

With respect to race, gender, and age, our results are similar to those published by Foreman et al who reported finding hospitalizations more frequently among females, older patients, and African Americans [6]. Sarcoidosis is generally more common in African Americans and women [16-18]. Also, African Americans and women tend to have more severe chronic disease and extrapulmonary involvement [3,4]. Differences in ascertainment and access to preventative and primary care may also be playing a role in this case of disparity of outcomes among African Americans [19]. Further, other investigators have reported that socioeconomic status plays a significant role in medical care and outcomes for patients with sarcoidosis [4]. In previous studies of sarcoidosis patients, less access to medical care has been associated with depression, and lower family income and lack of private or Medicare insurance is associated with increased severity of disease [20,21].

In contrast to previous work, however, we found that patients with insurance other than Medicaid actually had a higher increase in the rate of hospitalization compared to Medicaid patients. We speculate that this difference may be due to differential practice patterns or access to care/travel issues between patients with Medicaid and those with alternative insurance. For instance, those with insurance may be receiving more aggressive immunosuppression with corticosteroids, potentially more use of costly immunosuppressive alternatives to prednisone (with increased risky side effects), more diagnostic procedures, or may be able to more easily access care (whether geographically or financially), thereby influencing treatment and monitoring decisions by practitioners. Advancing radiographic imaging technology has also made it easier to find potential sarcoidosis cases and identify more subtle lung findings, which may have in turn led to more aggressive treatment approaches than in times of monitoring only with chest x-rays. Thus, the use of more sensitive imaging in insured populations may contribute to more aggressive immunosuppression.

Our hypothesis that the increase in hospitalizations is associated with more aggressive immunosuppressive treatments is further supported by the observation that hospitalizations with a primary sarcoidosis diagnosis are not increasing; increasing admission rates due to primary diagnoses other than sarcoidosis suggest that factors other than sarcoidosis itself are driving this trend. In addition, a recent retrospective study of healthcare utilization showed that sarcoidosis patients with greater corticosteroid use had a greater number of unscheduled emergency department visits [22]. Further support to our hypothesis is provided by the increasing body of literature showing that the widespread introduction of steroid-sparing immunosuppressive medications increases hospitalizations from other diseases, especially inflammatory bowel disease [23,24].

Because other studies have suggested a seasonal peak of diagnosis of sarcoidosis cases in the early spring, we investigated the seasonality of hospital admissions among patients with a sarcoidosis diagnosis [25,26]. Generally, seasonality of a disease or outcome implies that the driving force is an environmental influence, such as a seasonal viral infection or exposure. However, when we looked at patients specifically admitted for a primary diagnosis of sarcoidosis, we did not find any evidence of a seasonal pattern. We did find a seasonal pattern (peaking in January, February, and March) for admissions due to all causes. This could be associated with the timing of the influenza season. However, influenza cannot account for the dramatic eleven-year overall increase in the rate of hospitalizations of sarcoidosis patients. We performed further analyses to investigate whether the upward trend in sarcoidosis over the study period is still statistically significant after adjusting for influenza. This analysis was performed on the overall series and on the subseries comprised of patients 55 and older (data not shown). Specifically, we added a series representing national influenza activity as an explanatory variable in our seasonal ARIMA models for these series. In both models, the linear trend remains highly statistically significant (both p-values < 0.0001). Thus, adjustment for influenza does not impact the trend in sarcoidosis admissions.

There are several limitations to our study. First, a major limitation is that we use that we use administrative data, and do not have access to laboratory, radiographic or medication data. Second, we cannot confirm our hypothesis that immunosuppressive drugs are driving the change in epidemiology, nor can the coded diagnosis of sarcoidosis be confirmed. We also cannot report the burden of repeated hospitalizations. Due to the sampling design in the AHRQ database, there are no individual identifiers that can link patients across multiple admissions or over multiple years. Third, it is possible that medical advances are sustaining patients for longer, leading to increased number of hospitalizations that are not necessarily related to the severity or treatment of sarcoidosis. Fourth, it is also possible that subgroups with limited access to outpatient care (whether for financial or geographic reasons) may be admitted more frequently. Fifth, racial data in large databases may be missing or inaccurate. Last, it is possible that the increase in hospitalizations is due to an increase in the prevalence of sarcoidosis during the study period. However, hospitalizations specifically for a primary diagnosis have remained stable, suggesting that increased incidence of sarcoidosis is not the driving factor for hospitalization. The incidence in populations in the United Kingdom and Australia (both with similar incidence data to the United States) show that the diagnosis of sarcoidosis is not increasing over time [27,28].

## Conclusion

This study highlights the need for heightened surveillance of sarcoidosis patients, as the requirement for inpatient care in this group is increasing. Our observations are important given the unknown implications of evolving diagnostic and treatment approaches over this period of time. Our results also point to a need for research investigating risk factors for hospitalization, including the effects of medications, co-morbidities, demographics, geographic patterns, socioeconomic status, and differing practice patterns.

## Abbreviations

AHRQ: Agency for healthcare research and quality; ACF: Autocorrelation function; ICD-9-CM: International classification of diseases, 9th revision, clinical modification; SARIMA: Seasonal autoregressive integrated moving average; U.S.: United States.

## Competing interests

The authors declare that they have no financial or non-financial competing interests.

## Authors’ contributions

AKG, the guarantor of the manuscript, contributed to the design of the study, was responsible for data collection and analyses, and contributed to the writing and revising of the first draft of the manuscript. MY was responsible for data analyses, and contributed to the writing and revising of the manuscript. FT was responsible for data analyses, and contributed to revising the manuscript. JEC contributed to the design of the study, data analyses, and to the writing and revising of the manuscript. PMP contributed to the design of the study, data collection and analyses, and to the writing and revising of the first draft of the manuscript. All authors read and approved the final manuscript.

## Research funding support

This work was supported by the National Institutes of Health, Grant 1KL2RR024980: Institute for Clinical and Translational Science, University of Iowa.

## Pre-publication history

The pre-publication history for this paper can be accessed here:

http://www.biomedcentral.com/1471-2466/12/19/prepub
